# Recognition Interface of the Thrombin Binding Aptamer Requires Antiparallel Topology of the Quadruplex Core

**DOI:** 10.3390/biom11091332

**Published:** 2021-09-09

**Authors:** Julia Svetlova, Makar Sardushkin, Natalia Kolganova, Edward Timofeev

**Affiliations:** 1 Engelhardt Institute of Molecular Biology, Russian Academy of Sciences, 119991 Moscow, Russia; natha2006@yandex.ru; 2 Federal Research and Clinical Center of Physical-Chemical Medicine, 119435 Moscow, Russia; j.i.svetlova@gmail.com; 3 Universal Scientific Education and Research Network (USERN), G-Quadruplexes as INnovative ThERApeutiC Targets (G4-INTERACT), University of Pavia, 27100 Pavia, Italy; 4 Department of Chemical and Pharmaceutical Technologies and Biomedical Products, Mendeleev University of Chemical Technology of Russia, 125047 Moscow, Russia; s_makar@mail.ru

**Keywords:** G-quadruplex, recognition interface, thrombin binding aptamer, quadruplex core, loops, modification

## Abstract

Recent advances in G-quadruplex (GQ) studies have provided evidence for their important role in key biological processes (replication, transcription, genome stability, and epigenetics). These findings imply highly specific interactions between GQ structures and cellular proteins. The details of the interaction between GQs and cellular proteins remain unknown. It is now accepted that GQ loop elements play a major role in protein recognition. It remains unclear whether and to what extent the GQ core contributes to maintaining the recognition interface. In the current paper, we used the thrombin binding aptamer as a model to study the effect of modification in the quadruplex core on the ability of aptamer to interact with thrombin. We used alpha-2′-deoxyguanosine and 8-bromo-2′-deoxyguanosine to reconfigure the core or to affect *syn*–*anti* preferences of selected dG-residues. Our data suggest that core guanines not only support a particular type of GQ architecture, but also set structural parameters that make GQ protein recognition sensitive to quadruplex topology.

## 1. Introduction

Intensive studies of DNA and RNA G-quadruplexes (GQs) in recent decades have revealed their doubtless role in transcriptional control [1,2], telomere function, and epigenetic events [3]. The functionality of many cellular proteins is implemented through their binding with GQ sites, as demonstrated for a number of transcription factors [4], the RTEL1 protein [5], some DNA methyltransferases [3], and nucleolin [6]. In GQs, two major structural elements, loops and the GQ core, may contribute to the formation of the recognition interface between proteins and noncanonical DNAs or RNAs. Although stacked G-tetrads are a key hallmark of this secondary structure, it is reasonable to hypothesize that the GQ core guanines contribute largely to the maintenance of GQ architecture rather than to the protein recognition interface. In contrast, loop nucleotides seem to play a primary role in protein recognition. In the case of the thrombin-binding aptamer (TBA), the well-studied model of GQ–protein interaction [7], the dominant role in the formation of the recognition interface is attributed to two of its three loops. TBA is a 15 nt G-rich oligonucleotide that folds into antiparallel GQ and binds thrombin with high affinity, exhibiting a K_d_ value in the nanomolar range. TBA inhibits thrombin-catalyzed polymerization of fibrinogen. Numerous structural studies of TBA and its analogues suggest the major role of the two TT loops in building a molecular recognition area between TBA and thrombin [8,9]. Nevertheless, in the G quartet proximal to the protein, an interaction between guanine G5 (O4) and thrombin residue Arg77 (NH2) was identified [8,9] in crystallographic studies. Guanines of the second G quartet, which is distant from the protein, and nucleotides of the TGT loop do not have direct contact with the protein. Studies of TBA variant with inverted 5′ G-tracts (mTBAs) have shown that aptamer with reconfigured core and different distribution of *syn*–*anti* dG-residues is able to bind and inhibit thrombin [10,11]. Modification of TBA at its distal end—in particular, adding a duplex module [7] behind the distant G-quartet—does not principally affect the protein–aptamer interface. Moreover, particular variants of this type of modification are highly beneficial for the anticoagulant properties of the aptamer [7].

To further explore the effect that alterations in the GQ core may induce with respect to the recognition interface, we studied a series of TBA aptamers containing modifications of the core guanines. Two types of modifications, alpha-2′-deoxyguanosine (α-dG) and 8-bromo-2′-deoxyguanosine (Br-dG), were used to reconfigure the core or to affect *syn*–*anti* preferences within a particular G-quartet. Additionally, we composed an aptamer with one canonical and two modified G-quartets. We estimated an effect of modifications in the core on the folding topology, thermodynamic stability, and ability of the particular aptamer variant to bind and inhibit thrombin. Our data strongly suggest that a canonical antiparallel configuration in the two quartets proximal to the protein surface is required to maintain the functional recognition interface.

## 2. Materials and Methods

### 2.1. Oligonucleotides

DNA oligomers were synthesized by using an ABI 3400 DNA/RNA synthesizer and purified by reverse-phase high-performance liquid chromatography. Propargyl controlled pore glass (CPG) was purchased from Lumiprobe. Modified phosphoramidites were purchased from ChemGenes. Fluorescent aptamers were prepared by click reaction using 3′-propargyl-modified oligonucleotides and Sulfo-Cy5 azide (Lumiprobe).

### 2.2. Ultraviolet Thermal Denaturation

Absorbance versus temperature profiles were obtained with a Cary 50 spectrophotometer equipped with a Peltier cell holder. Melting experiments were performed at 295 nm in 10 mM sodium cacodylate (pH 7.2) and 100 mM KCl or 100 mM NaCl. The heating/cooling rate was 0.5 °C/min. The melting points were determined from derivative plots of the melting curves. The oligonucleotide concentration was in the range of 5 to 6 μM.

### 2.3. Circular Dichroism Spectroscopy

Circular dichroism (CD) measurements were performed at 20 °C with an aptamer concentration of 5 μM in 10 mM sodium cacodylate (pH 7.2) and 100 mM KCl or 100 mM NaCl by using a Chirascan CD spectrometer (Applied Photophysics). Before measurement, the aptamers were slowly annealed from 95 to 20 °C in the selected buffer.

### 2.4. Native Gel-Electrophoresis

Unlabeled aptamers (0.75 nmol) were annealed in 10 mM sodium cacodylate (pH 7.2) and 100 mM KCl or 100 mM NaCl in D_2_O and analyzed in a 20% native polyacrylamide gel (19:1) at 20 °C in 1× TBE buffer containing 10 mM KCl or 10 mM NaCl. The bands were visualized by UV shadowing.

### 2.5. Binding of Aptamers with Thrombin

Cy5-labeled aptamers were slowly annealed in 10 mM sodium cacodylate (pH 7.2) and 100 mM KCl in D_2_O. Thrombin (24 pmol) was added to 10 μL of an aptamer solution (1 μM), and the mixture was incubated for 30 min at 25 °C. An analysis of binding was carried out in a 12% native polyacrylamide gel (19:1) at 20 °C in 1× TBE buffer containing 10 mM KCl. Fluorescent bands of bound and free aptamers were visualized by using a research custom-made imaging system.

### 2.6. Fibrinogen Clotting in the Presence of Aptamers

Research-grade human thrombin from plasma was purchased from Renam (Moscow, Russia). Human thrombin (50 μL, 20 U/mL) was added to a solution of fibrinogen (2 mg/mL, Sigma-Aldrich, Munich, Germany) and aptamer (30 nM) in 1 mL of PBS in a quartz cuvette in a temperature-controlled cuvette holder of a spectrophotometer at 25 °C. Monitoring of the absorbance at 360 nm started immediately after the addition of thrombin and was stopped after the curve reached a plateau. The blank clotting curve was determined by measuring the absorbance in the absence of aptamer.

## 3. Results and Discussion

### 3.1. Aptamers with α-dG in the GQ Core

Previously, we showed that the GQ core of TBA may be reconfigured to form a unimolecular antiparallel structure with identical polarities in the two G-quartets [12] (variant A12 in Table 1). This effect could be induced by selective modification of nucleotides G1, G2, G10, and G11 with an alpha-anomeric analogue of dG. In contrast to the parent TBA, the circular dichroism (CD) spectrum of A12 features a positive band at 265 nm, thus mimicking parallel folding. Due to modification, variant A12 has lost the ability to bind and inhibit thrombin. To verify whether any combination of natural and modified thymine residues in TT loops in A12 may restore affinity to thrombin, we prepared new A12 variants with reconfigured GQ cores (Table 1). New A12 variants contained non-natural alpha-T residues at selected positions in TT loops. Designation of modified oligonucleotides refers to the configuration of thymidines in positions 3, 4, 12, and 13. Oligonucleotides were tagged at their 3′ end with propargyl linker to facilitate fluorescent labeling via click reaction with commercially available sulfo-Cy5 azide (Lumiprobe). Fluorescent variants of modified oligonucleotides were used for the detection of binding with thrombin in gel-shift assays. The structure of propargyl linker is shown in Supplementary Materials Figure S1.

**Figure 1:**
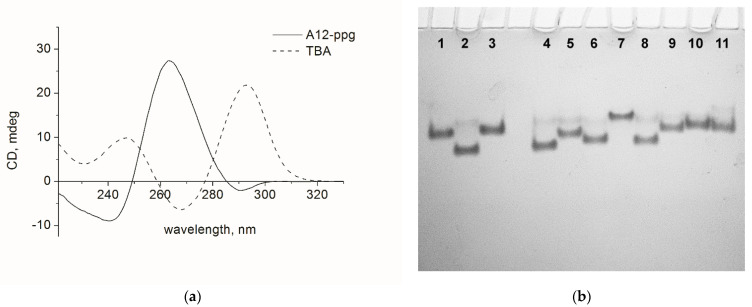
(**a**) CD spectra of the aptamers A12-ppg and TBA at 20 °C in 10 mM sodium cacodylate (pH 7.2) and 100 mM KCl. (**b**) Native polyacrylamide gel electrophoresis of the A12 aptamers in the presence of 10 mM KCl at 20 °C. (1) TBA, (2) A12, (3) TBA-ppg, (4) A12-ppg, (5) A12-βααβ, (6) A12-βαβα, (7) A12-αβαβ, (8) A12-αββα, (9) A12-α2β2, (10) A12β2α2, and (11) A12-α4.

The positive band at 265 nm in the CD spectra of modified aptamers in 100 mM KCl at 20 °C demonstrates that all new A12 variants retained the unusual configuration of guanines in the core (Figure 1a and Supplementary Materials Figure S2). With a single exception (A12-αβαβ), chimeric GQs showed a well-defined reversed UV-melting profile at 295 nm (Supplementary Materials Figure S3) typical for GQ structures. Aptamer A12-αβαβ exhibited an insignificant hypochromic change of absorbance at 295 nm, which most likely should be associated with the formation of a partially folded or misfolded structure. In the thermal difference spectra of A12 variants (Supplementary Materials Figure S4), A12-αβαβ appeared non-typical for the alpha-anomeric family, thus pointing to structural differences from other family members or insignificant contribution of pseudoparallel topology to the overall fold. Data on the thermal stability of alpha-chimeras indicate that A12-type structures are less stable than canonical antiparallel TBA (51 °C [12]). Combinations of alpha and beta nucleotides in TT loops in A12 series lead to variations in *T*_m_ values, due to more or less efficient stacking of loop thymines with an adjacent G-quartet.

Insignificant difference between A12 and A12-ppg in *T*_m_ values and CD patterns suggests that the presence of propargyl unit at the 3′ end of A12 series oligonucleotides does not affect the folding and stability of pseudoparallel GQs. We further verified the heterogeneity of folding of chimeric GQs. Oligonucleotides were annealed in 100 mM KCl buffer and analyzed at 20 °C in native polyacrylamide gel electrophoresis in the presence of 10 mM KCl (Figure 1b). All A12 variants were characterized by a single major band of varying mobility, supplemented by minor species migrating at a lower rate. Due to low stability of pseudoparallel 2-layer GQs, it is reasonable to suppose that minor bands are associated with an unfolded state. Variant A12-αβαβ migrates as a single band in the unfolded species area, thus suggesting that low K^+^ concentration in gel matrix (10 mM) does not support folded conformation of this oligonucleotide.

To estimate the ability of the A12 variants to bind thrombin, oligonucleotides bearing the 3′-propargyl unit were reacted with sulfo-Cy5 azide via a click reaction. Inspection of Cy5-A12 variants in native gel electrophoresis revealed an unusual effect that a fluorescent dye induces in GQs. The proportion of unfolded species in all samples was notably increased, including the reference TBA-ppg (Supplementary Materials Figure S5). Importantly, TBA variant labeled with Cy5 NHS ester through a 3′-aminolink (C7-aminolink, Glen Research), taken as an additional control, did not induce the formation of a large fraction of the unfolded aptamer (Supplementary Materials Figure S6a). Similar to cyanine dye, FAM azide also induced the formation of two species when conjugated with TBA-ppg. Therefore, the specific effect we observed can only be attributed to the propargyl linker. Indeed, due to the presence of a rigid amide unit in the linker structure, two configurations of the Cy5 tag at the 3′ end are possible, one of which may interfere with GQ folding.

In binding experiments, fluorescent aptamers were annealed in 100 mM KCl buffer and incubated with an excess of thrombin at 25 °C. An analysis of binding was performed in a 12% native polyacrylamide gel. The results of binding experiments show (Supplementary Materials Figure S6) that the combination of natural and modified thymidines in TT loops was not beneficial for restoring the recognition interface. Specific binding was observed only for the unmodified TBA control. It is worth noting that only the lower band of TBA-ppg-Cy5 was bound by excess thrombin.

To provide independent evidence that the modified A12 variants lack affinity for thrombin, we examined their anticoagulant properties in a fibrinogen clotting time test. Thrombin-induced fibrin gel formation in phosphate-buffered saline was monitored by the increase in absorbance at 360 nm due to light scattering. For comparative purposes, we estimated the time required to reach 50% of the absorbance maximum (t_1/2_). The clotting time analysis results are shown in Table 1. In the A12 family, the t_1/2_ values were close to the blank sample, indicating that alpha-chimeras do not inhibit fibrin polymerization. The cumulative data for chimeric alpha-TBA variants suggest that the configuration of the G-quartet in pseudo-parallel alignment is incompatible with the functional interface for thrombin recognition.

### 3.2. Aptamers with Br-dG in the Core

Non-natural Br-dG is known as a *syn*-biased modification and has been reported to cause 5‘-terminal G-tetrad polarity inversion in the parallel MYC quadruplex [14]. In the context of TBA modifications, Br-dG was shown to improve affinity and increase stability when incorporated into *syn* dG positions G1 and G10 [15]. More recently, single and double substitutions with Br-dG revealed that placing this modification in *anti*-dG positions induces diverse effect on thermal stability and affinity to thrombin [13]. Importantly, the antiparallel fold of the TBA was not affected in any case. In the present study, we verified the effect of Br-dG at *anti* positions or both (*anti* and *syn*) positions in G-tracts 2/4, 1/3, and 2/4. The designations of the corresponding Br-dG-modified oligonucleotides include the number of Br-dG residues and the G-tract numbers. Two modified aptamers, namely 2Br(1/2) and 2Br(1/3), were studied previously [13] and were taken as controls. New modified variants were used to examine whether tandem incorporation of a *syn*-favoring analogue may cause *anti*–*syn* rearrangement in the GQ core similar to that observed in A12 anomeric variants.

**Figure 2:**
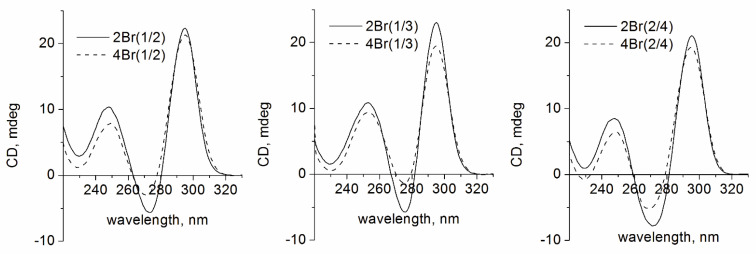
CD spectra of the Br-dG-modified aptamers at 20 °C in 10 mM sodium cacodylate (pH 7.2) and 100 mM KCl.

CD spectra of the oligonucleotides in 100 mM KCl at 25 °C showed that the canonical TBA-like antiparallel fold (Figure 2) was preserved in all modified variants. Studies of the thermal stability revealed a notable destabilization effect for 2Br(2/4) and 2Br(1/2) variants and a considerable increase in *T*_m_ for all 4Br variants (Table 1). The difference in the stabilities between 2Br and 4Br TBA variants is consistent with the *syn*–*anti* configuration of respective dG residues in the parent TBA. Modifications of *anti*-dG positions with Br-dG caused marked destabilization in 2Br(2/4) and 2Br(1/2). The effect of Br-dG modification was negligible in 2Br(1/3). Further substitutions at *syn*-positions in the same G-tracts increased the thermal stability up to a *T*_m_ value, which is approximately 10 °C higher in 4Br(2/4) and 4Br(1/2) than that in TBA (Table 1 and Supplementary Materials Figure S7). In 4Br(1/3), the stabilization effect was even higher (*T*_m_ = 71 °C). Thus, adding two additional modified residues to *syn*-positions induced a notable overbalance in terms of thermodynamic stability. This effect is most likely attributed to the additional stacking effect that the Br substituent may induce in modified structures 4Br. Importantly, modifications with Br-dG did not result in *anti*-to-*syn* conversion in any case.

**Figure 3:**
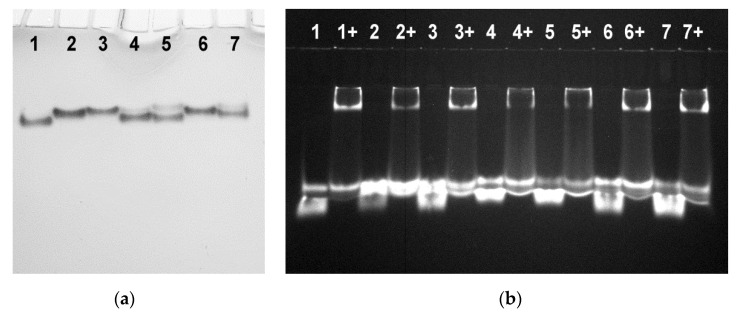
(**a**) Native polyacrylamide gel electrophoresis of the Br-dG-modified aptamers in the presence of 10 mM KCl at 20 °C: (1) TBA-ppg, (2) 2Br(2/4), (3) 4Br(2/4), (4) 2Br(1/3), (5) 4Br(1/3), (6) 2Br(1/2), (7) 4Br(1/2). (**b**) Native gel-electrophoresis image of the labeled 2Br and 4Br aptamers alone and in the presence of 2.4 equivalent thrombin (12% polyacrylamide gel, 20 °C, 10 mM KCl): (1) TBA-ppg-Cy5, (2) 2Br(2/4)-Cy5, (3) 4Br(2/4)-Cy5, (4) 2Br(1/3)-Cy5, (5) 4Br(1/3)-Cy5, (6) 2Br(1/2)-Cy5, and (7) 4Br(1/2)-Cy5. Symbol (+) indicates added thrombin.

Analysis of folding of Br-dG-modified aptamers in native gel electrophoresis revealed the formation of additional minor bands for aptamers 2Br(1/3), 4Br(1/3), and 4Br(1/2) (Figure 3a). It is unclear whether these minor bands can be associated with the unfolded state, since the thermal stabilities of the 4Br variants are relatively high. The only plausible reason for making this assumption is the presumable influence of the propargyl linker.

As expected for TBA analogues with antiparallel topology, the ability of the modified variants to bind thrombin was retained (Figure 3b). Binding with thrombin was confirmed by the shift of the lower bands of the labeled aptamers in polyacrylamide gel electrophoresis. In clotting time studies, inhibitory effect of particular 2Br or 4Br variant correlated with the arrangement of modified nucleotides rather than thermal stability. Thus, the pair 2Br(2/4) and 4Br(2/4) appeared to be the most potent inhibitors as compared to other Br-dG analogues (Table 1). The aptamers 2Br(1/2) and 4Br(1/2) showed somewhat lower activity. In the pair 2Br(1/3) and 4Br(1/3), the former had no inhibitory activity, and the latter showed a t_1/2_ value insignificantly exceeding the blank sample. Poor anticoagulant activity of aptamers clearly correlates with the arrangement of Br-dG residues in *anti* positions in the quartet proximal to the protein surface. Stabilization by adding Br-dG at *syn* positions in the 4Br series does not recover anticoagulant activity. This observation suggests that the aptamer recognition interface is very sensitive to changes induced in the GQ core and especially in the proximal G-quartet.

### 3.3. Modifying the Distant Tetrad and Adding the Third Modified Tetrad

The GQ core in the A12 chimeric series contained the entire G tract modified with α-dG analogues. Due to the inability of alpha nucleotides to adopt the *syn* configuration, it was possible to design a model with two identical G-quartets in the core. Similar polarity in both tetrads is responsible for the parallel-type CD pattern in alpha chimeras. In terms of the *syn*–*anti* configuration, modification of the selected dG residue with an alpha-analogue is equivalent to the ultimate alignment of the respective base in a *syn* position. Thus, it is possible to mimic the natural TBA configuration by substituting positions 1 and 10 in the distant tetrad with alpha-dG residues. With the aim of further modification of TBA with the third tetrad, it seemed reasonable to configure it in the same manner as the second modified tetrad. Following this strategy, we expected to retain the original alignment of guanines in the two G-quartets proximal to the protein. Adding the third tetrad with a configuration identical to the second tetrad would result in a three-layer hybrid G-quadruplex with a canonical recognition interface.

We prepared two TBA analogues with two and three guanines in each G-tract. Oligonucleotide TBA2α contained two substitutions for α-dG at positions 1 and 10. Three-layer aptamer 3Q4α was designed to have 5′-α,α,β consecutive dG residues in the first and third G-tracts. For comparison purposes, we included into study the oligonucleotide Q3 with three consecutive natural guanines in each G-tract. Its 3′ tagged version 3Q-ppg was also prepared for subsequent labeling with sulfo-Cy5 azide. It was previously shown that the natural three-layer prototype 3Q and closely related sequences form a predominantly parallel structure with propeller loops in the presence of K^+^. In the Na^+^ buffer, this oligonucleotide forms an antiparallel GQ [16,17,18,19]. Due to cation-dependent structural variations, three-layer QGs have been studied in both K^+^ and Na^+^ buffers.

**Figure 4:**
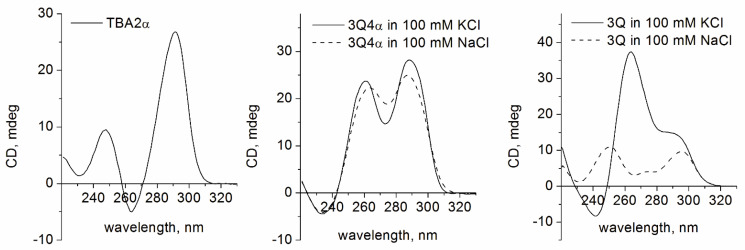
CD spectra of the modified variants TBA2α, 3Q4α, and 3Q at 20 °C in 10 mM sodium cacodylate (pH 7.2) and 100 mM KCl or 100 mM NaCl (3Q4α and 3Q).

The CD spectrum of TBA variant TBA2α in 100 mM KCl was typically antiparallel, whereas 3Q4α showed a classical hybrid fold in both 100 mM KCl and 100 mM NaCl (Figure 4). The presence of α-dG residues at positions 1 and 10 in analogue TBA2α appeared detrimental to the stability of GQ, showing a *T*_m_ value of 26 °C in 100 mM KCl (Table 1 and Supplementary Materials Figure S8). Surprisingly, three-layer variant 3Q4α showed extremely high stability with a *T*_m_ of 70.4 °C in 100 mM NaCl. In K^+^ buffer, the *T*_m_ value could not be determined due to the high stability of the formed structure (Supplementary Materials Figure S8). Natural oligonucleotide 3Q and its 3′-modified variant showed a mixture of parallel and antiparallel structures in 100 mM KCl and two-phase melting transition (Figure 4; Supplementary Materials Figures S8 and S9). In Na^+^ buffer, natural 3Q variants formed antiparallel structures (Figure 4 and Supplementary Materials Figure S9).

**Figure 5:**
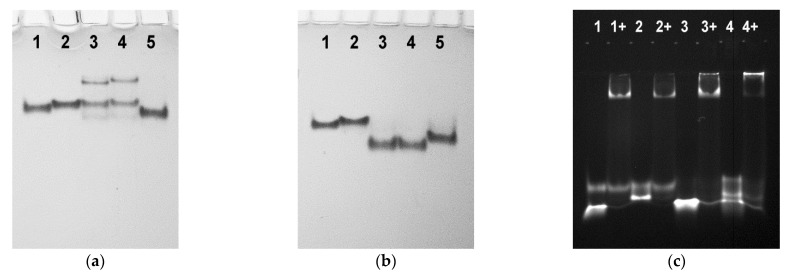
(**a**,**b**) Native polyacrylamide gel electrophoresis of the modified variants TBA2α, 3Q4α, 3Q, and 3Q-ppg at 20 °C in the presence of 10 mM KCl (**a**) or 10 mM NaCl (**b**). (1) TBA-ppg, (2) TBA2α, (3) 3Q, (4) 3Q-ppg, and (5) 3Q4α. (**c**) Native gel-electrophoresis image of the labeled aptamers TBA2α-Cy5, 3Q4α-Cy5, and 3Q-ppg-Cy5 alone and in the presence of 2.4 equivalent thrombin (12% polyacrylamide gel, 20 °C, 10 mM KCl). (1) TBA-ppg-Cy5, (2) TBA2α-Cy5, (3) 3Q4α-Cy5, and (4) 3Q-ppg-Cy5. Symbol (+) indicates added thrombin.

Native gel electrophoresis revealed a notable difference between the 3Q4α and 3Q aptamers (Figure 5a,b). The three-layer alpha aptamer formed a single structure in both K^+^ and Na^+^ buffers. In contrast, in the presence of KCl, the 3Q variants appeared in the gel as a set of multiple bands representing presumably a parallel monomer, a parallel dimer, and a concomitant antiparallel structure. In the presence of Na^+^ ions, the 3Q variants were represented by a single band.

As proposed, the two models, TBA2α and 3Q4α, retained the canonical recognition interface and the ability to bind thrombin (Figure 5c). The binding with thrombin was observed for the both labeled modified variants, as detected by gel electrophoresis in native 12% polyacrylamide gel. Natural variant Cy5-3Q also showed protein binding. However, in this case, the fluorescent band was observed at the very beginning of the lane, which indicates the formation of a multi-species complex. Interestingly, despite this fact, 3Q and 3Q-ppg were not active in the fibrinogen clotting test. In contrast to the natural prototype 3Q, the three-layer 3Q4α analogue appeared to be the most efficient anticoagulant in a series of studied modified aptamers.

Our data on the ability of modified TBAs to retain the protein-recognition interface in response to modifications in the core strongly support the idea of preserving both the TT loop and the proximal tetrad configuration intact. In the A12 series of modified TBA variants, neither configuration of loop nucleotides was able to restore aptamer affinity to the protein. In an antiparallel architecture, modifications affecting the proximal G-quartet are most damaging to the recognition interface. Modifications in the second G-quartet, although not crucial for the thrombin–aptamer interface, may strongly affect the protein recognition. Configuration of the third modified G4 layer also does not prevent binding to the protein but again may notably affect the affinity and anticoagulant characteristics. It is worth noting that adding the third modified tetrad resulted in a considerable increase in the stability of non-natural TBA. In this respect, TBA variants with an additional distant G4 layer represent a promising model for the rational design of GQ aptamers, as long as such modification does not induce structural polymorphism.

The results of our study suggest that the protein–GQ interaction is affected by the proximal G-quartet configuration. Therefore, core guanines not only contribute to the overall type of GQ folding but also establish parameters of interactions that make protein–GQ recognition sensitive to a particular G4 topology.

## Figures and Tables

**Table 1:** Modified TBA variants.

ID	Sequence ^1^	CD Type (K^+^)	*T*_m_, °C (K^+^) ^2^	t_1/2_, min ^4^
TBA	GGTTGGTGTGGTTGG	antiparallel	51 [12]	5.1
TBA-ppg	GGTTGGTGTGGTTGG-ppg	antiparallel	49.6	4.9
A12	ggTTGGTGTggTTGG	parallel	38.9 [12]	1.1
A12-ppg	ggTTGGTGTggTTGG-ppg	parallel	39.4	1.0
A12-α4	ggttGGTGTggttGG-ppg	parallel	35.4	1.1
A12-βαβα	ggTtGGTGTggTtGG-ppg	parallel	40.2	1.0
A12-αβαβ	ggtTGGTGTggtTGG-ppg	parallel	nd	0.9
A12-βααβ	ggTtGGTGTggtTGG-ppg	parallel	39.8	1.1
A12-αββα	ggtTGGTGTggTtGG-ppg	parallel	43.9	1.1
A12-α2β2	ggttGGTGTggTTGG-ppg	parallel	37.5	1.0
A12-β2a2	ggTTGGTGTggttGG-ppg	parallel	34.6	0.9
2Br(2/4)	GGTTGBTGTGGTTGB-ppg	antiparallel	37.2	3.5
4Br(2/4)	GGTTBBTGTGGTTBB-ppg	antiparallel	60.3	3.6
2Br(1/3)	GBTTGGTGTGBTTGG-ppg	antiparallel	51.5; 53.2 [13]	1.1
4Br(1/3)	BBTTGGTGTBBTTGG-ppg	antiparallel	71.0	1.3
2Br(1/2)	GBTTGBTGTGGTTGG-ppg	antiparallel	42.8; 40.8 [13]	1.8
4Br(1/2)	BBTTBBTGTGGTTGG-ppg	antiparallel	63.5	1.7
TBA2α	gGTTGGTGTgGTTGG-ppg	antiparallel	26 ^3^	1.6
3Q	GGGTTGGGTGTGGGTTGGG	mixed	47.0 and 76.6	1.0
3Q-ppg	GGGTTGGGTGTGGGTTGGG-ppg	mixed	45.0 and 74.6	0.9
3Q4α	ggGTTGGGTGTggGTTGGG-ppg	hybrid	nd	3.8

^1^ g = α-dG, t = α-T, B = Br-dG, ppg = propargyl linker; ^2^ by UV at 295 nm; ^3^ estimate; ^4^ t_1/2_ for blank sample was 1.0 min; Δ*T*_m_ = ±0.5 °C; Δt_1/2_ = ±0.1 min.

## Data Availability

Not applicable.

## Supplementary Materials

The following are available online at https://www.mdpi.com/article/10.3390/biom11091332/s1. Figure S1: Structure of the propargyl linker. Figure S2: CD spectra of modified A12 aptamer variants at 20 °C in 10 mM sodium cacodylate (pH 7.2) and 100 mM KCl. Figure S3: UV melting profiles of modified A12 aptamer variants at 295 nm in 10 mM sodium cacodylate (pH 7.2) and 100 mM KCl. Figure S4: Thermal difference spectra (90 °C vs. 20 °C) of the aptamer series A12 in 10 mM sodium cacodylate (pH 7.2) and 100 mM KCl. Figure S5: (a) Denaturing (7M urea) gel-electrophoresis image of the labeled A12 aptamers in 20% polyacrylamide gel at 50 °C: (1) TBA-ppg-Cy5, (2) A12-ppg-Cy5, (3) A12-βααβ-Cy5, (4) A12-βαβα-Cy5, (5) A12-αβαβ-Cy5, (6) A12-αββα-Cy5, (7) A12-α2β2-Cy5, (8) A12-β2α2-Cy5, (9) A12-α4-Cy5, and (10) TBA-C7-Cy5 (C7 aminolink). (b) Native gel-electrophoresis image of the labeled A12 aptamers in 20% polyacrylamide gel at 20 °C in the presence of 10 mM KCl: (1) TBA-ppg-Cy5, (2) A12-ppg-Cy5, (3) A12-βααβ-Cy5, (4) A12-βαβα-Cy5, (5) A12-αβαβ-Cy5, (6) A12-αββα-Cy5, (7) A12-α2β2-Cy5, (8) A12-β2α2-Cy5, and (9) A12-α4-Cy5. Figure S6: Native gel-electrophoresis image of the labeled A12 aptamers alone and in the presence of 2.4 equivalent thrombin (12% polyacrylamide gel, 20 °C, 10 mM KCl). (a) (1) TBA-ppg-Cy5, (2) A12-ppg-Cy5, (3) A12-βααβ-Cy5, (4) A12-βαβα-Cy5, (5) A12-αβαβ-Cy5, (6) TBA-C7-Cy5 (C7 aminolink). (b) (1) TBA-ppg-Cy5, (2) A12-αββα-Cy5, (3) A12-α2β2-Cy5, (4) A12-β2α2-Cy5, and (5) A12-α4-Cy5. Symbol (+) indicates added thrombin. Figure S7: UV melting profiles of the Br-dG-modified aptamers at 295 nm in 10 mM sodium cacodylate (pH 7.2) and 100 mM KCl. Figure S8: UV melting profiles of the modified variants TBA2α, 3Q4α, 3Q, and 3Q-ppg at 295 nm in 10 mM sodium cacodylate (pH 7.2) and 100 mM KCl or 100 mM NaCl (3Q4α, 3Q, and 3Q-ppg). Figure S9: CD spectra of the aptamer 3Q-ppg at 20 °C. Figure S10: Normalized clotting curves.
