# Supplementary material for: Recognition Interface of the Thrombin Binding Aptamer Requires Antiparallel Topology of the Quadruplex Core
Source: Biomolecules. 2021 Sep 9;11(9):1332. doi: 10.3390/biom11091332 (PMC8471065; doi:10.3390/biom11091332)
Supplement: Supplementary file 1 [file biomolecules-11-01332-s001.zip › biomolecules-1318797-supplementary.pdf]

Supplementary Information

Recognition Interface of the Thrombin Binding Aptamer Requires Anti-Parallel Topology of the Quadruplex Core

Julia Svetlova et. al.

Supplementary Figures

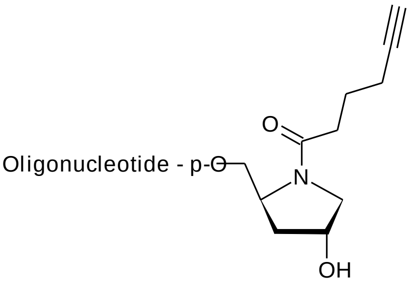

Figure S1. Structure of the propargyl linker.

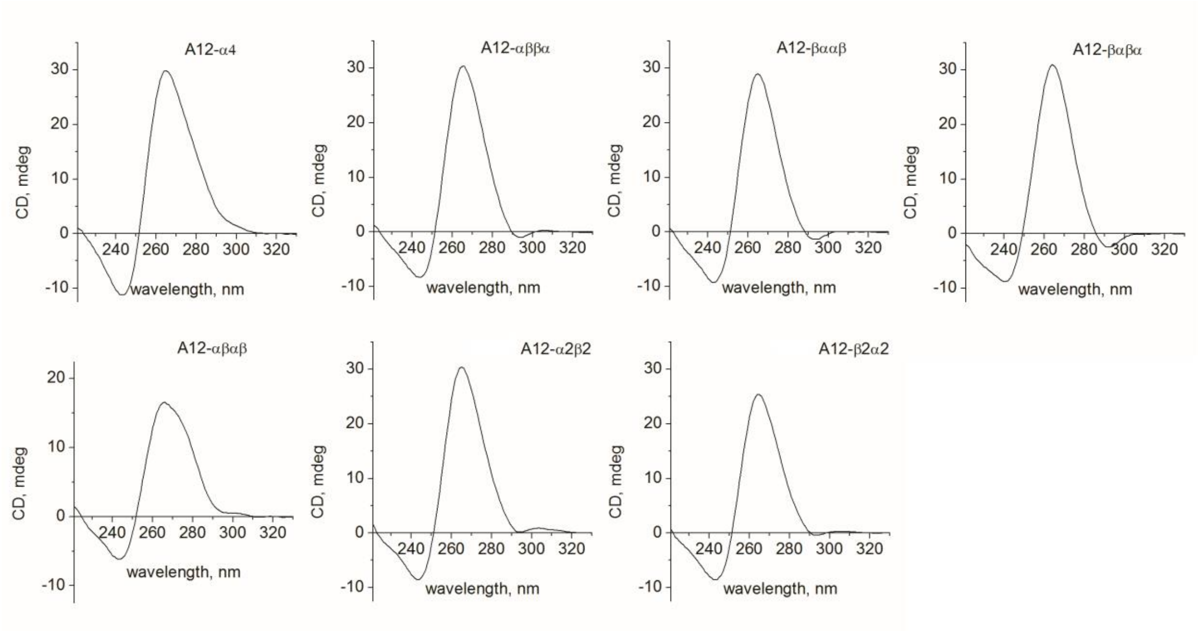

Figure S2. CD spectra of modified A12 aptamer variants at 20°C in 10 mM sodium cacodylate (pH 7.2) and 100 mM KCl.

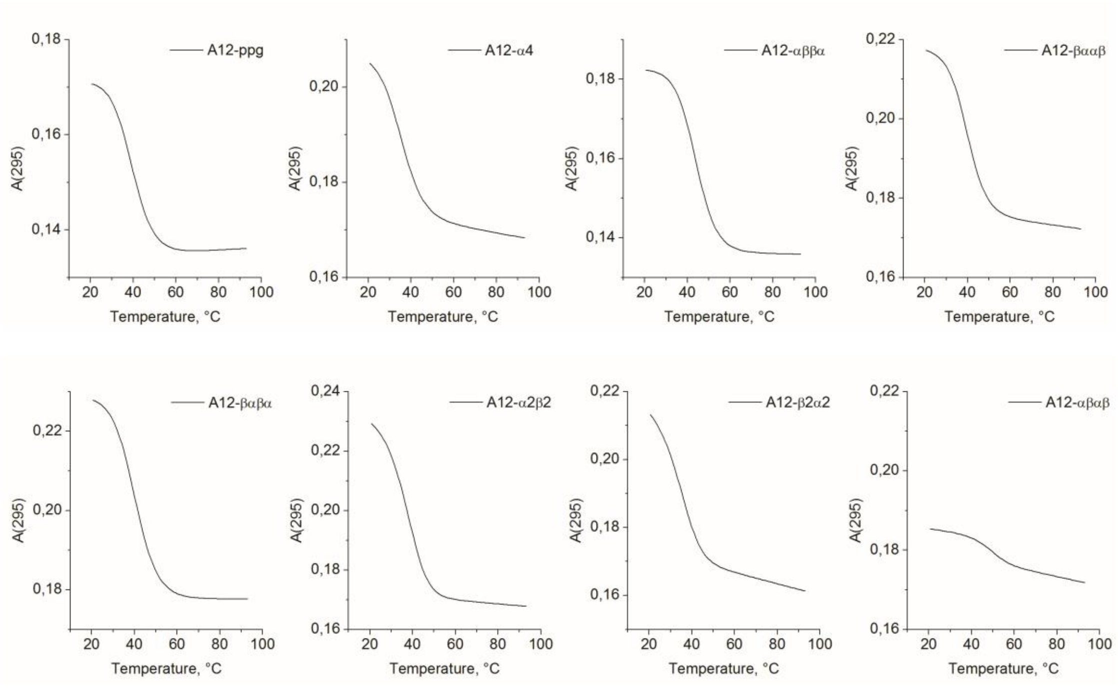

Figure S3. UV melting profiles of modified A12 aptamer variants at 295 nm in 10 mM sodium cacodylate (pH 7.2) and 100 mM KCl.

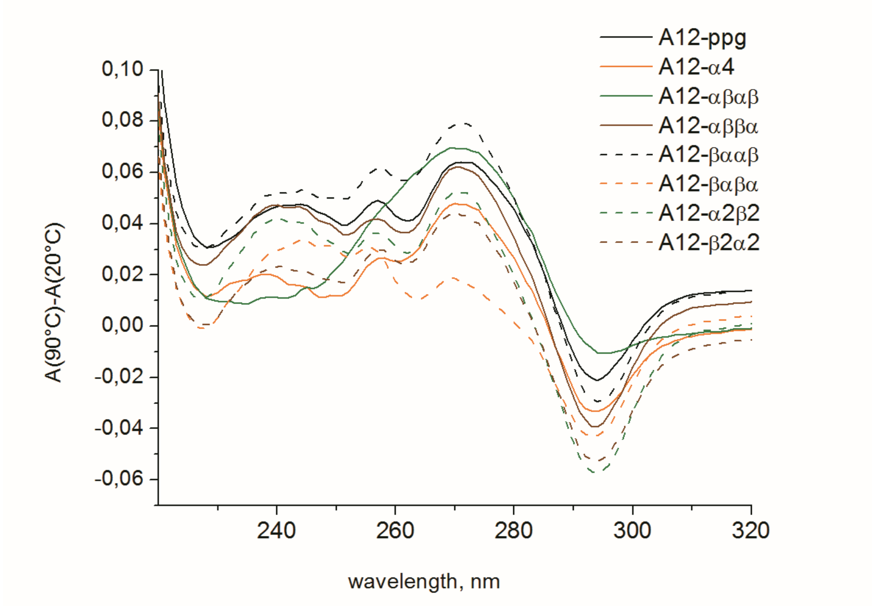

Figure S4. Thermal difference spectra (90°C vs. 20°C) of the aptamer series A12 in 10 mM sodium cacodylate (pH 7.2) and 100 mM KCl.

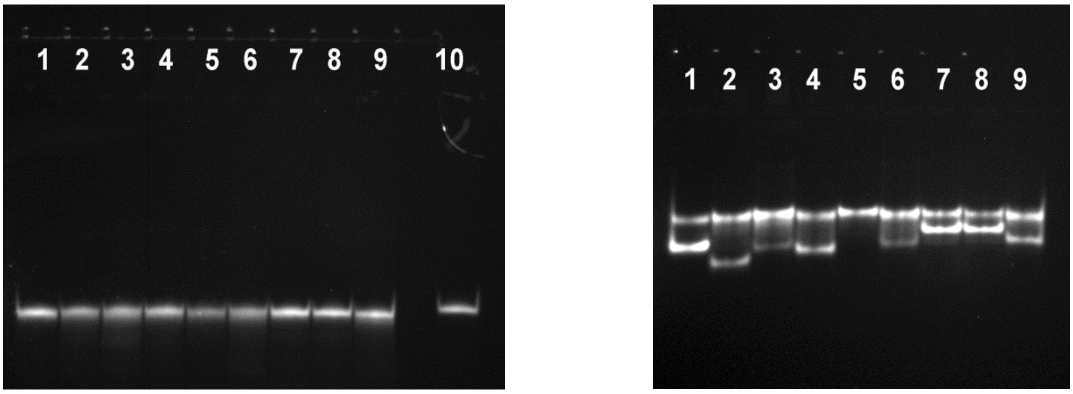

(a) (b)

Figure S5. (a) Denaturing (7M urea) gel-electrophoresis image of the labeled A12 aptamers in 20% polyacrylamide gel at 50°C. (1) – TBA-ppg-Cy5, (2) – A12-ppg-Cy5, (3) – A12-βααβ-Cy5, (4) – A12-βαβα-Cy5, (5) – A12-αβαβ-Cy5, (6) – A12-αββα-Cy5, (7) – A12-α2β2-Cy5, (8) – A12-β2α2-Cy5, (9) – A12-α4-Cy5, (10) – TBA-C7-Cy5 (C7 aminolink). (b) Native gel-electrophoresis image of the labeled A12 aptamers in 20% polyacrylamide gel at 20°C in the presence of 10 mM KCl. (1) – TBA-ppg-Cy5, (2) – A12-ppg-Cy5, (3) – A12-βααβ-Cy5, (4) – A12-βαβα-Cy5, (5) – A12-αβαβ-Cy5, (6) – A12-αββα-Cy5, (7) – A12-α2β2-Cy5, (8) – A12-β2α2-Cy5, (9) – A12-α4-Cy5.

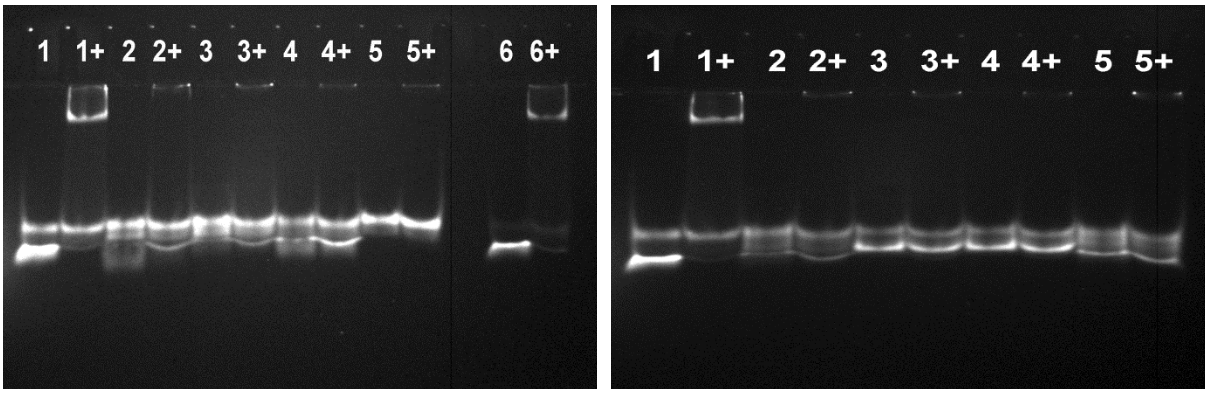

(a) (b)

Figure S6. Native gel-electrophoresis image of the labeled A12 aptamers alone and in the presence of 2.4 equivalent thrombin (12% polyacrylamide gel, 20°C, 10 mM KCl). (a) (1) – TBA-ppg-Cy5, (2) – A12-ppg-Cy5, (3) – A12-βααβ-Cy5, (4) – A12-βαβα-Cy5, (5) – A12-αβαβ-Cy5, (6) – TBA-C7-Cy5 (C7 aminolink). (b) (1) – TBA-ppg-Cy5, (2) – A12-αββα-Cy5, (3) – A12-α2β2-Cy5, (4) – A12-β2α2-Cy5, (5) – A12-α4-Cy5. Symbol (+) indicates added thrombin.

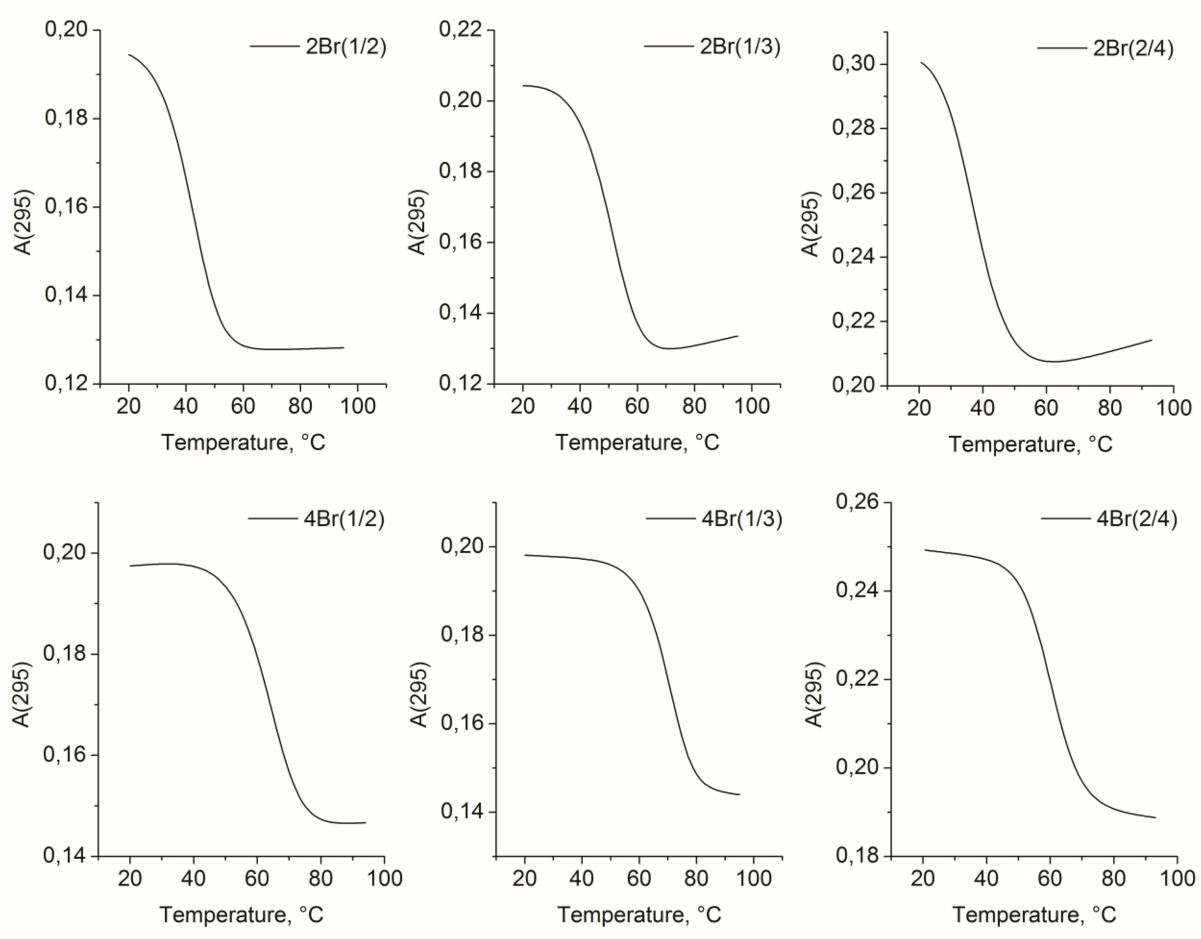

Figure S7. UV melting profiles of the Br-dG-modified aptamers at 295 nm in 10 mM sodium cacodylate (pH 7.2) and 100 mM KCl.

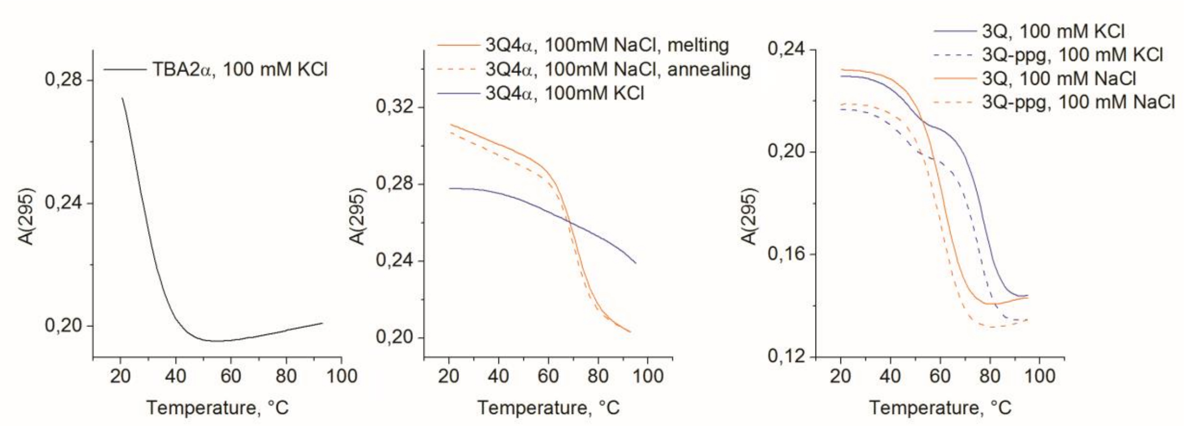

Figure S8. UV melting profiles of the modified variants TBA2α, 3Q4α, 3Q and 3Q-ppg at 295 nm in 10 mM sodium cacodylate (pH 7.2) and 100 mM KCl or 100 mM NaCl (3Q4α, 3Q, and 3Q-ppg).

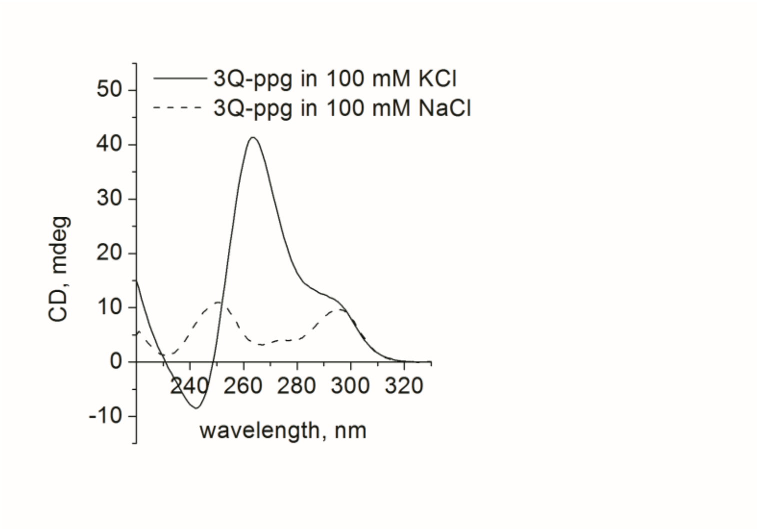

Figure S9. CD spectra of the aptamer 3Q-ppg at 20°C.

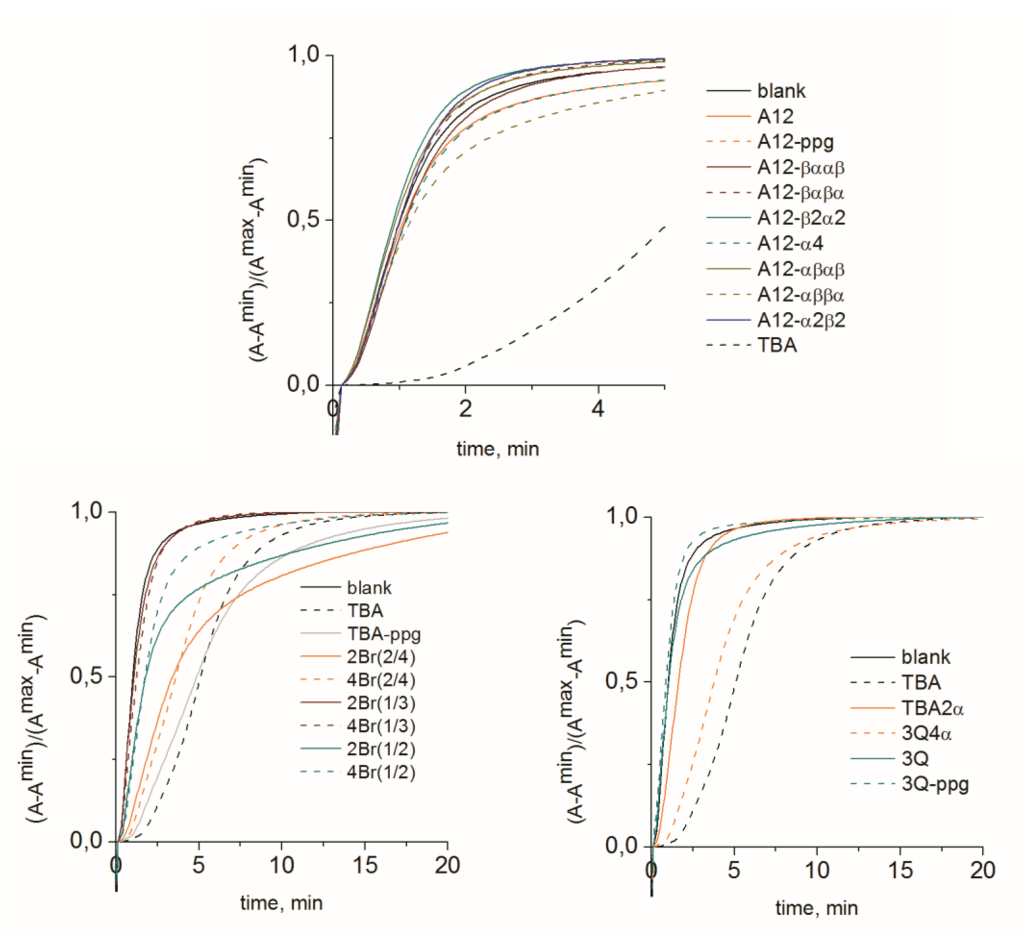

Figure S10. Normalized clotting curves.
